# Molecular Characterization of Dengue Virus Type 1 in Zhejiang in 2019

**DOI:** 10.3389/fcimb.2021.673299

**Published:** 2021-10-05

**Authors:** Wenwu Yao, Zhangnv Yang, Xiuyu Lou, Haiyan Mao, Hao Yan, Yanjun Zhang

**Affiliations:** Department of Microbiology, Zhejiang Provincial Center for Disease Control and Prevention, Hangzhou, China

**Keywords:** dengue virus, phylogentic analysis, E gene, amino acid mutation, prevention

## Abstract

Dengue fever (DF) is a mosquito-borne viral disease caused by the dengue virus (DENV), which is considered one of the most important arboviruses in the world. This study aimed to determine the molecular, epidemiological, and phylogenetic characterization of 174 DENV-1 (132 indigenous cases and 42 imported cases) isolated from nine municipalities of Zhejiang province in 2019. The analyses of phylogenetics, haplotypes, and amino acid substitutions were conducted based on the full envelope (E) gene sequences. Sixty-four haplotypes were clustered into two main clades, with isolates from Wenzhou and Taizhou mainly clustered into clade I and Hangzhou and Ningbo cases clustered into clade II. Six sites of amino acid substitutions including A88T, F96L, M297V, T339S, I378L, and V436I were only observed in strains isolated from Ningbo and Hangzhou, while two sites of amino acid substitutions including V312L and V380I were observed in strains from Taizhou and Wenzhou. In our study, strains were in high homology with the strains from Southeast Asian countries, thus cases in Zhejiang were probably imported from Southeast Asian countries. The strains from different regions in Zhejiang were clustered in the same branch which may be caused by the continuous import of cases in the same country at different time periods. After the continuous outbreak in Zhejiang province, some sites of the dengue gene have mutated, and the effects need further study.

## Introduction

Dengue is one of the most globally prevalent arboviruses in the word, which is caused by the dengue virus (DENV). DENV belongs to the genus *Flavivirus* of the family *Flaviviridae* (Ngwe Tun et al., 2016). Human infection with DENV is through a bite of mosquito, and the clinical symptoms range from asymptomatic infection to dengue hemorrhagic fever (DHF), dengue shock syndrome (DSS), and death (Cruz et al., 2013; Martins et al., 2014). The World Health Organization (WHO) has estimated that approximately 50–100 million dengue infections occur every year, of which 500,000 cases are DHF and 22,000 deaths. The genome of DENV is a single-stranded positive-sense RNA and approximately 11,000 nucleotides. Its genome encodes three structural proteins including C (capsid), prM/M (precursor of membrane), and envelope (E); seven nonstructural proteins, including NS1, NS2A, NS2B, NS3, NS4A, NS4B, and NS5. DENV are represented by four distinct serotypes (DENV-1, DENV-2, DENV-3, and DENV-4) (Wang et al., 2000; Conceição et al., 2010; Lee et al., 2012), and each of the serotypes can be subclassified into several genotypes on the basis of their E gene sequences (Li et al., 2017). Many Asian countries including Singapore, Malaysia, Pakistan, India, Japan, China, and Indonesia have also reported outbreaks of DENV (Jarman et al., 2008; Holmes et al., 2009; Vu et al., 2010; Akram and Idrees, 2016; Tajima et al., 2017). Since the first reported outbreak of DENV in 1978 in China, Southeastern coastal provinces, including Guangdong, Fujian, Zhejiang, and Hainan have been affected by DENV outbreaks, where the genotypes of DENV-1, DENV-2, DENV-3, and DENV-4 were detected (Wang et al., 2009; Xiong and Chen, 2014; Chen and Liu, 2015; Sun et al., 2017).

Zhejiang province, with a population of 56 million, is one of the important members of the Yangtze River Delta. Meanwhile, as the important entry/exit port province in east China, Zhejiang has frequent exchanges and cooperation with foreign countries, especially with Southeast Asian countries, where DENV originated. Plains, hills, and mountains constitute the complex geographical features of Zhejiang, and the complex environments provide ideal conditions for the growth of mosquitoes, which is the vector for the transmission of DENV (Guo et al., 2014). In 2004, a DENV outbreak in Cixi of Zhejiang caused 83 people to get infected 18]. More cases occurred in Yiwu of Zhejiang in 2009, and 196 cases of DF were reported (Sun et al., 2011). In 2017, a historical outbreak occurred in Zhejiang province, and a total of 1,229 cases were reported, with 93.8% emerging in Hangzhou (Yan et al., 2018). In recent years, DF cases have been reported almost every year in Zhejiang.

Despite that the four serotypes of DENV have been detected in Zhejiang in recent years, the serotype 1 has gradually become the dominant serotype. Therefore, in our study, 174 cases of DENV-1 (including 132 indigenous cases and 42 imported cases) were used to conduct a molecular investigation and epidemiological and phylogenetic characterization of DENV that were detected during the dengue outbreak in Zhejiang province in 2019.

## Materials and Methods

### Sample Collected

Serum samples for this study were kept in the Zhejiang provincial Centers for Disease Control and Prevention (CDC) in 2019. DV nucleotide detection and serotype identification of all DF cases were determined according to diagnostic criteria for DF (WS216-2008) of the Ministry of Health of China, and 174 cases of DENV-1 were collected in this study.

### Virus Strains

DENV strains in our study were isolated from the positive serums of patients, and the *Aedes albopictus* gut cell line (C6/36) was used for virus propagation. Serum samples were diluted 10-fold with fresh MEM medium (Gibco, Waltham, MA, USA) and then inoculated with a C6/36 cell monolayer, which was incubated in MEM medium supplemented with 2% fetal bovine serum (FBS, Gibco, USA) at 28°C for 5–7 days. Cells showed typical cytopathic effects (CPE) being considered DENV positive. These culture supernatants were then stored at −80°C for use.

### RNA Extraction and Sequencing

The RNeasy Mini Kit (Qiagen, Hilden, Germany) was used to extract virus RNA from 200 µl cell culture supernatants according to the manufacturer’s instructions. RT-PCR was conducted using the PrimeScript™ One Step RT-PCR Kit (TaKaRa, Shiga, Japan). The full-length E genes of the 174 DENVs were amplified with the following primers: DV1-E-F (5′CAAGAACCGAAACRTGGATGTC3′) and DV1-E-R (5′GGCTGATCGAATTCCACACAC3′). The 50-μl PCR reaction included 25 μl of 1× one-step buffer, 2 μl of PrimeScript one-step enzyme mix, 1 μl of each primer (20 μM), 4 μl of the template RNA, and 17 μl of RNase Free dH2O. RT-PCR reverse transcription reaction was initiated at 50°C for 30 min, then denaturation at 94°C for 2 min, followed by 40 cycles of denaturation step at 94°C for 30 s, and primer annealing at 53°C for 30 s, primer extension at 72°C for 2 min, and a final extension step at 72°C for 10 min. The PCR products were purified by agarose gel electrophoresis, and then sequenced by Sangon Biotech Co. (Shanghai, China).

### Data Analysis

One hundred seventy-four sequences from the isolated strains were spliced in SEQMAN from the LaserGene package (DNASTAR Inc., Madison, WI, USA), and 39 reference sequences were downloaded from the GenBank database. The nucleotide sequences were aligned using the professional software Clustal W. The phylogenetic tree based on full E gene sequences was constructed by using the neighbor-joining method with the Tajima-Nei model in MEGA v.6.06. The bootstrap value was 1,000 replicates, and only values >70% are presented. Haplotype analyses for the 174 full E gene sequences were conducted using the DNAsp5.1 software. Then the sequences were divided into nine groups (Wenzhou/Hangzhou/Jinhua/Taizhou/Ningbo/Shaoxing/Jiaxing/Huzhou/Zhoushan) according to the geographic location, and haplotype network was made by Popart 1.7 software. The differences of amino acid sequence were analyzed by MEGA-x.

### Ethics Statement

The authors declare that they have no conflict of interest. This study was approved by the Institutional Ethics Committee of the Zhejiang Provincial Center for Disease Control and Prevention. Informed oral consent was obtained from all of the patients recruited for the study.

## Results

### Case Confirmation and Epidemiology of Dengue in 2019 in Zhejiang

**Figure 1:**
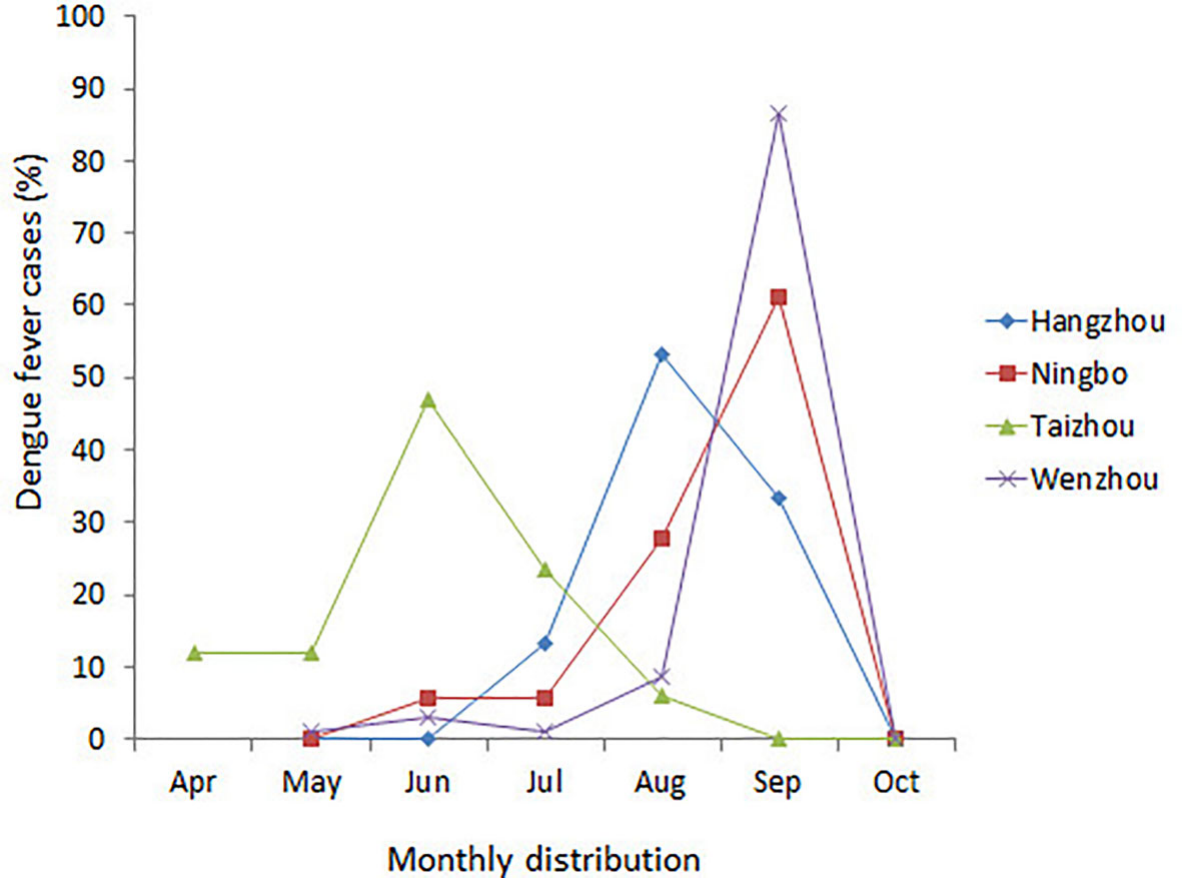
Monthly distribution of dengue fever cases in four main cities in Zhejiang Province, China, 2019.

A total of 174 DENV-1 serum samples from nine municipal Centers for Disease Control and Prevention (CDC) of Zhejiang, including Wenzhou, Hangzhou, Jinhua, Taizhou, Ningbo, Shaoxing, Jiaxing, Huzhou, and Zhoushan, were verified by multiplex RT-PCR. Geographical distribution of 132 indigenous cases and 42 imported cases are shown in **Table 1**, and the detailed geographical distribution of cases is shown in **Table 2**. Monthly distribution of dengue fever cases in four main cites in Zhejiang Province is shown in **Figure 1**. In Ningbo and Wenzhou, cases peak was in September, while cases peaks in Hangzhou and Taizhou were in August and June, respectively.

**Table 1:** Demographic characteristics of dengue cases in Zhejiang Province, 2019.

City	Indigenous	Imported	Total
Wenzhou	91	13	104
Hangzhou	15		15
Taizhou	8	16	24
Ningbo	10	8	18
Jinhua	3	2	5
Zhoushan	3		3
Jiaxing		3	3
Shaoxing	1		1
Hzhou	1		1
Total	132	42	174

**Table 2:** Distribution of countries of origin for imported dengue cases.

Original country	Cases number	Total
Cambodia	Taizhou: 004-014, 017,019,020,024; Wenzhou: 032,078,096-098,101; Jinhua: 004,005; Ningbo: 013,017; Jiaxing: 003	26
Thailand	Wenzhou: 060,062; Ningbo: 018; Jiaxing: 002	4
Malaysia	Taizhou: 002	1
Other cities in China	Wenzhou: 080-082, 087,089; Ningbo: 003,004,011,015,016; Jiaxing: 001	11

### Evolutionary Relationships of DENV-1 Haplotypes

**Figure 2:**
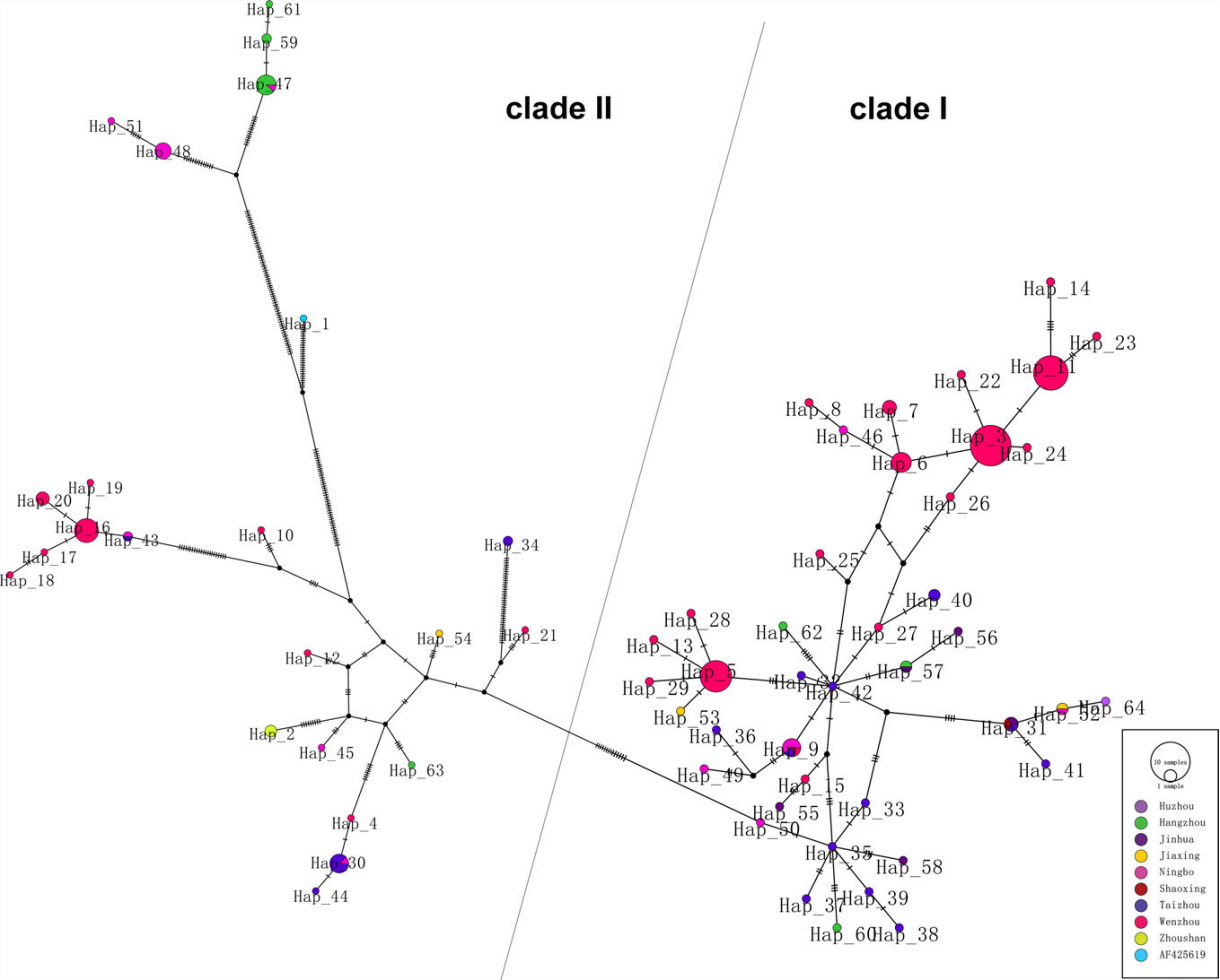
Phylogenetic networks of 174 DENV-1 strains. Each node in the network represents a haplotype, and the circle size of the node represents the number of sequences contained in this haplotype.

As shown in phylogenetic networks, the 64 haplotypes were clustered into two main clades (**Figure 2**). Nucleotide diversity was 0.03336, and haplotype diversity was 0.950. Forty-one haplotypes clustered into clade I and 23 haplotypes clustered into clade II. Clade I is mainly composed of Wenzhou and Taizhou cases, and clade II is mainly composed of Hangzhou and Ningbo cases. Hap_42 (TZ 004, imported from Cambodia), connected to Hap_5, Hap_9, Hap_27, and Hap_57, was supported as the potential ancestral haplotypes. The Hap_5 and Hap_35 were the other two core haplotypes. The long branches of Hap_61, Hap_59, Hap_47, Hap_51, and Hap_48 correspond to a significant mutation.

### Phylogenetic Analysis

**Figure 3:**
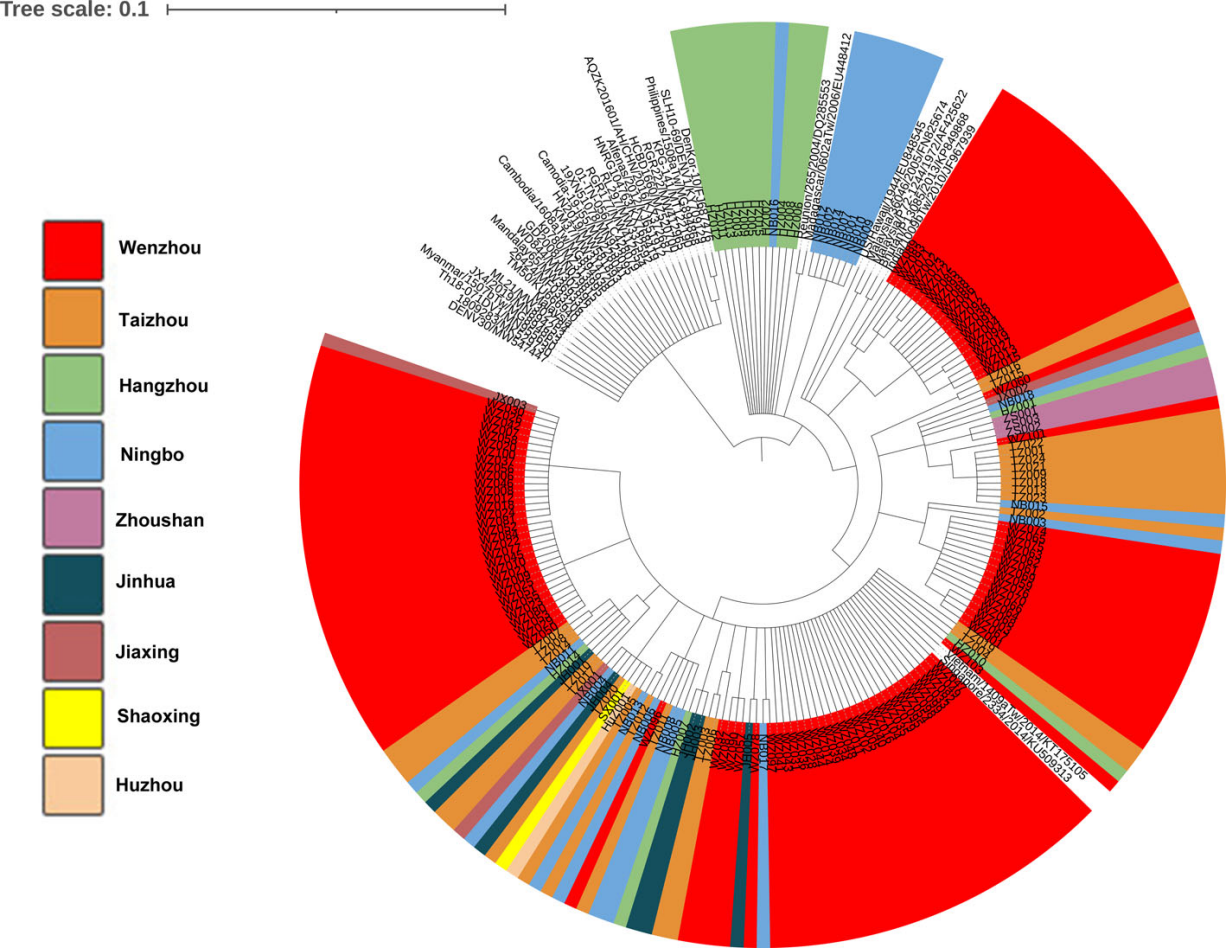
Phylogenetic analysis of isolated DENV-1 based on the E gene. Phylogenetic tree was constructed on the basis of whole nucleotide sequences of the E protein gene of DENV-1 showing the relationship of 174 DENV-1 isolated and 39 of reference sequences.

Phylogenetic tree was obtained based on the 174 of nucleotide sequences of the E gene of DENV-1and 39 of reference sequences (**Figure 3**). The results demonstrated that 73.3% of the strains (11/15) from Hangzhou were clustered in the same branch with 39% viruses (7/18) from Ningbo. Almost all the strains found in Taizhou were clustered with strains from Wenzhou. The strains in our study were in high homology with the strains from Vietnam/1409a Tw/2014 and Singapore/2334/2014. Phylogenetic analysis of strains found in Wenzhou (**Figure 3** shown by red color) was more complex, most of the strains were clustered with the two strains Vietnam/1409a Tw/2014 from Vietnam and Singapore/2334/2014 from Singapore in 2014, and some of the strains were clustered with the strains from Zhoushan, Ningbo or Taizhou.

### Amino Acid Substitutions

Mainly 10 sites of the E protein amino acid substitutions were observed among the DENV-1 strains compared with the D1 Hawaii reference strain (**Table 3**). Six sites of amino acid substitutions including A88T, F96L, M297V, T339S, I378L, and V436I were observed in strains isolated from Ningbo and Hangzhou, while two sites of amino acid substitutions including V312L and V380I were observed in strains found in Taizhou and Wenzhou. Amino acid substitutions in two sites (I161T and L402F) were almost observed among the strains isolated from 4 cities (Ningbo, Hangzhou, Taizhou and Wenzhou).

**Table 3:** Amino acid differences in the E protein of DENV-1 strains in comparison with the D1 Hawaii reference strain (AF 425619).

Position	88	96	161	297	312	339	378	380	402	436
D1 Hawaii AF425619	A	F	I	M	V	T	I	V	L	V
NB001	T	L	T	V	–	S	L	–	F	I
NB002	T	L	T	V	–	S	L	–	F	I
NB009	T	L	T	V	–	S	L	–	F	I
NB007	T	L	T	V	–	S	L	–	F	I
NB018	T	L	T	V	–	–	–	I	F	–
NB016	T	L	T	V	–	S	L	–	F	I
HZ002	T	L	T	V	–	S	L	–	F	I
HZ004	T	L	T	V	–	S	L	–	F	I
HZ003	T	L	T	V	–	S	L	–	F	I
HZ005	T	L	T	V	–	S	L	–	F	I
HZ006	T	L	T	V	–	S	L	–	F	I
HZ009	T	L	T	V	–	S	L	–	F	I
HZ008	T	L	T	V	–	S	L	–	F	I
HZ011	T	L	T	V	–	S	L	–	F	I
TZ011	–	–	T	–	L	–	–	I	F	–
TZ014	–	–	T	–	L	–	–	I	F	–
TZ020	–	–	T	–	L	–	–	I	F	–
TZ004	–	–	T	–	L	–	–	I	F	–
TZ005	–	–	T	–	L	–	–	I	F	–
TZ007	–	–	T	–	L	–	–	I	F	–
TZ012	–	–	T	–	L	–	–	I	F	–
TZ019	–	–	T	–	L	–	–	I	F	–
TZ017	–	–	T	–	L	–	–	I	F	–
WZ100	–	–	T	–	L	–	–	I	F	–
WZ096	–	–	T	–	L	–	–	I	F	–
WZ097	–	–	T	–	L	–	–	I	F	–
WZ098	–	–	T	–	L	–	–	I	F	–
WZ099	–	–	T	–	L	–	–	I	F	–
WZ102	–	–	T	–	L	–	–	I	F	–
WZ103	–	–	T	–	L	–	–	I	F	–
WZ104	–	–	T	–	L	–	–	I	F	–

## Discussion

Since 2004, DENV outbreak occurred in Cixi of Zhejiang and causing 83 people to get infected; cases of DF have been reported nearly every year in Zhejiang province (Xu et al., 2007). Especially in 2017, a large outbreak occurred in Zhejiang with a total of 1,229 DF cases being reported, as DENV-2 was the dominant serotype (Yan et al., 2018). However, DENV-1 has gradually become the dominant serotype since then. Zhejiang province was one of the important regions of international trade, commercial trade, and tourism; the convenient communication with the world has increased the incidence of DENV infections. In 2019, 174 cases of DENV-1 were collected from nine municipal CDC of Zhejiang, including 132 indigenous cases and 42 imported cases, with 26 of the imported cases being imported from Cambodia (**Table 2**), and the phylogenetic, molecular, and epidemiological characteristics were conducted in our study. The peak of the cases was almost in June, August, and October, which is consistent with the mosquito peak season. One hundred four out of 174 total cases (59.8%) and 13 out of 42 (31%) imported cases were come from Wenzhou. It is well known that Wenzhou is an important commercial city in China and located in the southeast of Zhejiang province. Meanwhile, Wenzhou has the ideal conditions for the life cycle of mosquitoes, including rivers, mountains, hills, and comfortable temperature. DENV is spread by mosquito bites, and the infection of DENV is closely related to the density of mosquitoes. Trade and population movements make it possible for the spread of the DENV. Those superior geographical location and frequent commercial exchanges provide conditions for the outbreaks of DENV infections.

Phylogenetic networks showed that most strains from Wenzhou and Taizhou clustered into the same clade, and more strains from Hangzhou and Ningbo clustered into another clade (**Figure 2**). The strains founded in Wenzhou differentiate two subgroups, and one subgroup only spreads within Wenzhou. By detecting the difference of amino acid, we found an interesting phenomenon: amino acid substitutions (A88T, F96L, M297V, T339S, I378L, and V436I) only occurred in strains from Ningbo and Hangzhou, while sites of amino acid substitutions (V380I and V312L) occurred in strains from Wenzhou and Taizhou. Whether the substitutions are related to virulence still need further study. The result was consistent with the phylogenetic networks. It may be that the DENV has spread to surrounding areas after circulating in one area in a period of time. Geographically, Wenzhou and Taizhou are adjacent, while Hangzhou and Ningbo are relatively close.

The E gene encodes envelope protein, which is responsible for cell receptor binding and the key target for neutralizing antibodies and vaccine development. The amino acid substitutions in the envelope protein may influence the recognition and binding of antibodies. In our study, the phylogenetic tree was obtained based on the full E gene. The result indicated that 20 indigenous strains from Wenzhou (WZ001–WZ003, WZ059, WZ061–WZ076) and two imported strains (Taizhou002 and Ningbo003) were clustered in the same subclade with strains from Singapore (SGEHID104009Y13) and Malaysia (TM45). It demonstrated that some early cases in Wenzhou may be imported from Southeast Asian countries. Eleven strains from Hangzhou (HZ002–HZ006, HZ008, HZ009, HZ011–HZ013, HZ015) and eight strains from Ningbo (NB001, NB002, NB007, NB009, NB010, NB012, NB014) were clustered with the strains from Madagascar/0602aTw/2006 and Reunion/265/2004.

We analyzed the molecular characterization, amino acid mutation, and phylogenetic tree of 174 DENV-1 strains in Zhejiang province in 2019. The strains from Hangzhou had a close link with the virus from Ningbo; however, the strains from Wenzhou were closely related to the isolates from Taizhou. The strains in Zhejiang province were probably imported from Southeast Asian countries. The strains from different regions in Zhejiang were clustered in the same branch which may be caused by the continuous import of cases in the same country at different time periods. After the continuous outbreak in Zhejiang province, some sites of the dengue gene have mutated, and the effects need further study.

## Data Availability Statement

The raw data supporting the conclusions of this article will be made available by the authors, without undue reservation.

## Ethics Statement

The studies involving human participants were reviewed and approved by Institutional Ethical Committee of Zhejiang Provincial Center for Disease Control and Prevention. Written informed consent for participation was not required for this study in accordance with the national legislation and the institutional requirements.

## Author Contributions

HY and YZ designed the study. HY and WY performed the data. ZY and WY analysed data. All authors contributed to the article and approved the submitted version.

## Funding

This study was supported by Natural Science foundation of Zhejiang Province( LGF20H260003), Health Leading Talents Program of Zhejiang Province (2018(22)), Key Disciplinary of Health and Family Planning Commission of Zhejiang Province (CX-9), and Medical Science and Technology Program of Zhejiang Province (2020RC050,2020KY092, 2018KY341).

## Conflict of Interest

The authors declare that the research was conducted in the absence of any commercial or financial relationships that could be construed as a potential conflict of interest.

## Publisher’s Note

All claims expressed in this article are solely those of the authors and do not necessarily represent those of their affiliated organizations, or those of the publisher, the editors and the reviewers. Any product that may be evaluated in this article, or claim that may be made by its manufacturer, is not guaranteed or endorsed by the publisher.
